# Supplementary material for: Vaccination boosts protective responses and counters SARS-CoV-2-induced pathogenic memory B cells
Source: medRxiv. 2021 May 12:2021.04.11.21255153. Originally published 2021 Apr 14. Preprint. [Version 2] doi: 10.1101/2021.04.11.21255153 (PMC8057254; doi:10.1101/2021.04.11.21255153)
Supplement: Supplement 1 [file media-1.pdf]

Fig. S1

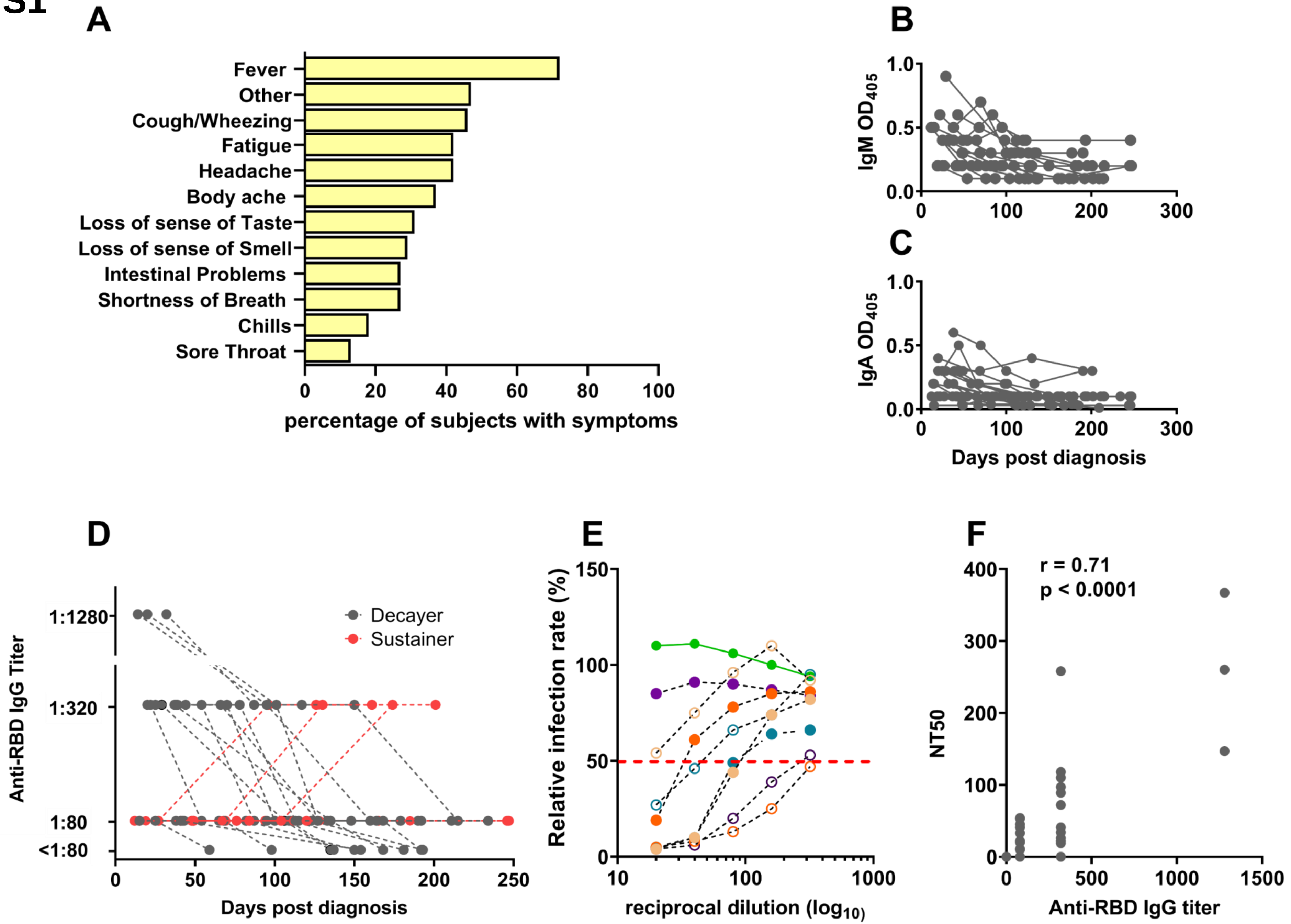
A
Fever
Other
Cough/Wheezing
Fatigue
Headache
Body ache
Loss of sense of Taste
Loss of sense of Smell
Intestinal Problems
Shortness of Breath
Chills
Sore Throat
0 20 40 60 80 100
percentage of subjects with symptoms
B
IgM OD405
1.0 0.5 0.0
0 100 200 300
C
IgA OD405
1.0 0.5 0.0
0 100 200 300
Days post diagnosis
D
Anti-RBD IgG Titer
1:1280
1:320
1:80
<1:80
Decayer
Sustainer
0 50 100 150 200 250
Days post diagnosis
E
Relative infection rate (%)
150 100 50 0
10 100 1000
reciprocal dilution (log10)
F
r = 0.71
p < 0.0001
NT50
400 300 200 100 0
0 500 1000 1500
Anti-RBD IgG titer

Supplementary Fig. 1. (A) Frequency of symptoms related to SARS-CoV-2 infection in study subjects (n = 83). Each row represents a (type of) clinical symptom(s) and shows the percentage of study subjects who self-reported that symptom(s). **(B,C**) **Anti-RBD levels of IgM and IgA antibodies.** Line graphs showing IgM and IgA antibody levels in the same subjects as in main **Fig 2B**. (**D**) **Titers of circulating anti-RBD IgG.** Trajectories of RBD-specific IgG antibody titers corresponding to the OD_{405} data in main **Fig. 1A.** Study subjects and definition of sustainers and decayers as per **Fig. 1B**. (**E**) **Method for neutralizing titer 50 (NT50) determination.** Representative neutralization curves demonstrating the determination of 50% neutralization (red dotted line) for the calculation of NT50 values presented in main **Fig. 1C** and main **Fig. 2C,D**. Relative infection rates were obtained by dividing the fluorescence readings (derived from fluorescently tagged virus) of the sample-treated wells by the un-treated control wells (infection rates above 100% result from small biological variations in untreated wells, and indicate no neutralization). The light green line corresponds to an uninfected (negative control) sample exhibiting no neutralizing activity. (**F**) **Correlation between anti-RBD titer and NT50.** Correlation analysis between anti-RBD IgG endpoint titers (x-axis) and neutralizing titers (y-axis) (n = 44). The Spearman's correlation coefficient r and corresponding p value were calculated.

Fig. S2

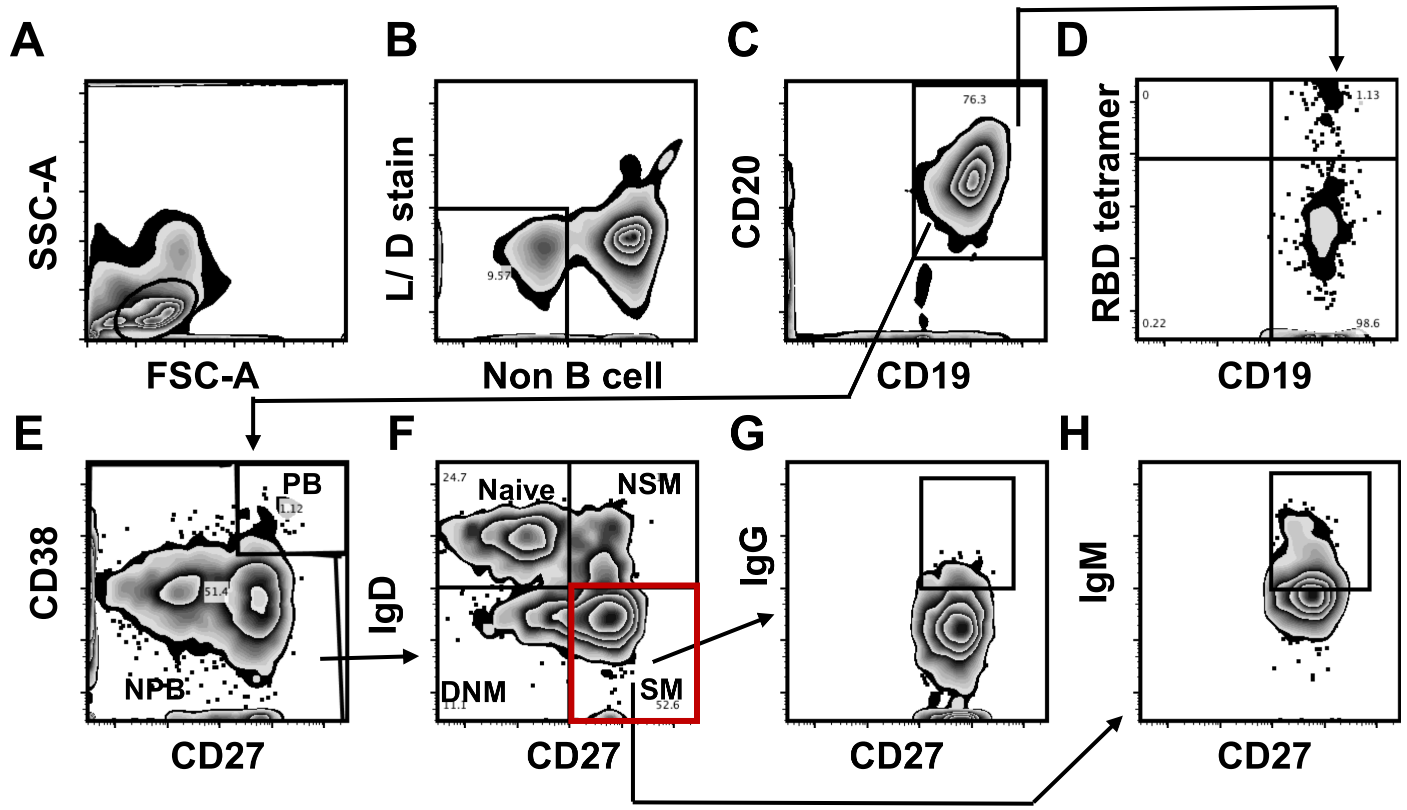

A
SSC-A
FSC-A
B
L/ D stain
9.57
Non B cell
C
CD20
76.3
CD19
D
RBD tetramer
0
1.13
0.22
98.6
CD19
E
CD38
PB
1.12
51.4
NPB
CD27
F
IgD
24.7
Naive
NSM
DNM
11.1
SM
52.6
CD27
G
IgG
CD27
H
IgM
CD27

Supplementary Fig. 2. Flow cytometry gating strategy for B cell subsets. (**A**) Physical parameters. (**B**) Exclusion of dead cells and non-B cells ($CD14^+$, $CD3^+$, $CD4^+$, $CD56^+$). (**C**) $CD19^+CD20^+$ cells (B cells) were further gated to distinguish (**D**) RBD-specific B cells utilizing fluorescently labeled RBD tetramers. (**E**) Plasmablast ($CD27^+CD38^{hi}$) and non plasmablasts. (**F**) Non-plasmablasts were further gated on CD27 and IgD for memory phenotyping: Naïve B cells ($CD27^-IgD^+$), Non switched memory B cells (NSM; $CD27^+IgD^+$), Switched memory B cells (SM; $CD27^+IgD^-$), and Double negative memory B cell (DNM; $CD27^-IgD^-$). Switched memory (SM) cell were further gated to determine the frequencies of (**G**) IgG^+ switched memory B cell (IgG^+SM) and (**H**) and IgM^+ switched memory B cell (IgM^+SM).

Table S1. Characteristics of study participants

Characteristics	n=83
Age (years), median (IQR*)	45 (31-56)
Gender, n (%)	
Male	38 (46)
Female	45 (54)
Race, n (%)	
African American or Black	6 (7)
Alaskan Native of American Indian	0 (0)
Asian or Pacific Islander	13 (16)
White	63 (76)
Other	1 (1)
Ethnicity, n (%)	
Hispanic or Latino	16 (19)
Non-Hispanic	67 (81)
SARS-CoV-2 PCR Positivity, n (%)	
Positive	81 (98)
Negative	0 (0)
Not performed**	2 (2)
Peak Disease Severity, n (%) [Female (F), Male (M)]	
Asymptomatic	1 (1) [0F, 1M]
Mild (Non-hospitalized; 1-4 symptoms)	55 (66) [30F, 25M]
Moderate (Non-hospitalized; 5 or more symptoms)	22 (27) [16F, 6M]
Severe (Hospitalized)	5 (6) [2F, 3M]
Days After Symptom Onset at Initial Collection (n=82***), median (IQR*)	34 (26-42)

*Interquartile range.
**Subjects who did not receive the PCR test were diagnosed by exposure history (PCR-positive household members), COVID-19 symptoms, and positive chest x-ray findings.
***The asymptomatic subject was not included.

Table S2. Characteristics of study subjects followed longitudinally

Characteristics	n=23
Age (years), median (IQR*)	39 (31-56)
Gender, n (%)	
Male	9 (39)
Female	14 (61)
Race, n (%)	
African American or Black	2 (9)
Alaskan Native of American Indian	0 (0)
Asian or Pacific Islander	3 (13)
White	18 (78)
Ethnicity, n (%)	
Hispanic or Latino	6 (26)
Non-Hispanic	17 (74)
SARS-CoV-2 PCR Positivity, n (%)	
Positive	21 (91)
Negative	0 (0)
Not performed**	2 (9)
Peak Disease Severity, n (%) [Female (F), Male (M)]	
Asymptomatic	1 (4) [0F, 1M]
Mild (Non-hospitalized; 1-4 symptoms)	15 (65) [6F, 9M]
Moderate (Non-hospitalized; 5 or more symptoms)	7 (31) [5F, 2M]
Severe (Hospitalized)	0 (0) [0F, 0M]
Days After Symptom Onset at Initial Collection (n=22***), median (IQR*)	38.5 (26-42)

*Interquartile range.
**Subjects who did not receive the PCR test were diagnosed by exposure history (PCR-positive household members), COVID-19 symptoms, and positive chest x-ray findings.
***The asymptomatic subject was not included.

Table S3. Comparison of cell subsets in non-infected and infected subjects

Cell subset	Phenotype	A, Non-infected	B, Infected <2m post	C, Infected >5m post	p value	
					A vs. B [i]	B vs. C [ii]
Total B Cell	$CD19^+$ $CD20^+$	9.6 (7.0-12.0)	6.0 (4.0-10.7)	6.4 (4.8-8.1)	0.08	0.36
PB	$CD38^{hi}$ $CD27^+$	0.4 (0.2 - 0.7)	1.1 (0.4-1.6)	0.4 (0.2-0.9)	0.01	0.04
Naïve	$CD27^-$ IgD^+	55 (42.2-59.7)	34.9 (25.9-34.8)	30.2 (18.1-35.8)	0.0004	0.04
NSM	$CD27^+$ IgD^+	11 (6.9-16.2)	12.1 (7.0-18.3)	9.7 (2.8-14.6)	0.27	0.04
SM	$CD27^+$ IgD^-	23.4 (21.3-29.3)	36.2 (26.6-51.6)	35 (28.2-43.5)	0.006	0.25
SM IgM^+	$CD27^+$ IgD^- IgM^+	14.9 (9.2-22.3)	32.7 (22.8-36.6)	10.1 (7.7-17.3)	0.001	<0.0001
SM IgG^+	$CD27^+$ IgD^- IgG^+	6.7 (2.0-8.0)	4.3 (3.5-5.9)	7.7 (2.7-14.9)	0.21	0.04
DNM	$CD27^-$ IgD^-	7.9 (5.0-12.5)	10.4 (7.5-16.4)	21.3 (12.2-34.6)	0.08	0.003
RBD^+ B Cell	$CD19^+$ $CD20^+$ RBD^+	ND	1.13 (0.88-1.36)	1.03 (0.8-1.62)	NA	0.08
RBD^+ Naïve	RBD^+CD27^- IgD^+	ND	50.9 (40.6-68.7)	50 (43.3-62.0)	NA	0.30
RBD^+ NSM	RBD^+ $CD27^+$ IgD^+	ND	5.4 (3.3-14.3)	8.6 (5.3-14.2)	NA	0.4
RBD^+ SM	RBD^+ $CD27^+$ IgD^-	ND	27.1(14.1-39.3)	21.4 (13.2-35.6)	NA	0.42
RBD^+ DNM	RBD^+ $CD27^-$ IgD^-	ND	7.4 (4.4-11.1)	11.8 (6.5-18.2)	NA	0.01

Data are presented as median and interquartile range (IQR); PB - Plasmablast; NSM - non switched memory; SM - switched memory; DNM - double-negative memory

[i]Comparison between non-infected and infected (<2 months) using Mann-Whitney U test

[ii]Comparison between infected (<2 months) and infected (>5 months) using Wilcoxon matched pairs test

ND, Non detected

NA, Not applicable

Table S4. Comparison of cell subsets in pre- and post-vaccinations of non-infected and infected subjects

Cell subset	Phenotype	A, Infected, pre-vac	B, Infected, post-vac	C, Non-infected, pre-vac	D, Non-infected, post-vac	p value	
						A vs. B [i]	C vs. D [ii]
B Cell	$CD19^+$ $CD20^+$	5.1 (3.3-7.2)	3.5 (2.2 -4.7)	12.3 (11.5-16.7)	4.3 (3.2-4.8)	0.03	0.004
RBD^+ B Cell	$CD19^+$ $CD20^+$ RBD^+	0.5 (0.3-0.8)	1.1 (0.9-1.6)	ND	0.8 (0.5-0.9)	0.003	0.004
PB	$CD38^{hi}$ $CD27^+$	0.6 (0.1-1.1)	0.4 (0-1.2)	0.4 (0.06-0.9)	0.2 (0.2-0.6)	0.31	0.34
Naïve	$CD27^-$ IgD^+	25.8 (12.5-37.1)	63.3 (5.4-71.7)	57.7 (49.1-63.6)	66.5 (64.1-73.9)	0.002	0.01
NSM	$CD27^+$ IgD^+	5.7 (0.4-10.6)	3.5 (2.7-9.3)	9.7 (9.0-11.4)	5.4 (3.7-8.9)	0.42	0.01
SM	$CD27^+$ IgD^-	41.7 (25.1-49.7)	14.9 (12.5-17.7)	23.4 (20.2-30.6)	14.9 (12.5-17.7)	0.002	0.01
DNM	$CD27^-$ IgD^-	19.2 (13.3-49.8)	14.7 (9.8-17.8)	9.9 (2.9-10.6)	9.8 (6.1-12.5)	0.007	0.27

Data are presented as median and interquartile range (IQR); PB - Plasmablast; NSM - non switched memory; SM - switched memory; DNM - double negative memory; vac - vaccination

Comparisons of infected pre- vs post-vaccination[i] and non-infected pre- vs post-vaccination[ii] using Wilcoxon matched pairs test
